# Successful Superselective Renal Artery Embolization for the Treatment of Active Renal Bleeding and Large Hematoma after Nephropyelolithotomy

**DOI:** 10.5334/jbr-btr.825

**Published:** 2015-09-15

**Authors:** S. Akay, V. Akgun, B. Karaman, F. Ors

**Affiliations:** 1 Department of Radiology, Sirnak Military Hospital, Sirnak, Turkey; 2 Department of Radiology, Gulhane Military Medical School, Ankara, Turkey

A 22-year-old man who had had percutaneous nephropyelolithotomy seven days previously, was referred to our department for urinary ultrasonography (US), because of high fever (38.5°C) and increased WBC (17700/µL). Following the detection of hyperechogeneities in the dilated collecting system and parenchyma of the right kidney on US, a non-enhanced computed tomography (CT) was planned with the suspicion of bleeding. On CT, large homogeneous-hyperdense areas filling the middle part and lower pole of the kidney primarily suggesting active renal bleeding and hematoma, were seen (Figs. A and B). Because of the progressive decrease in the hemoglobin level one day later, catheter angiography was performed to determine the probable bleeding focus and perform endovascular treatment at the same time, if required. On catheter angiography, contrast extravasation was seen at the subsegmentary arteries in the middle section and inferior pole of the right kidney causing large hyperdense areas on corresponding CT images (Figs. C and D). After superselective diagnostic study of the bleeding small arteries, embolization with nonspherical material was performed without complication. On control angiogram, it was confirmed that the bleeding had stopped (Fig. E).

**Figures A–E:**
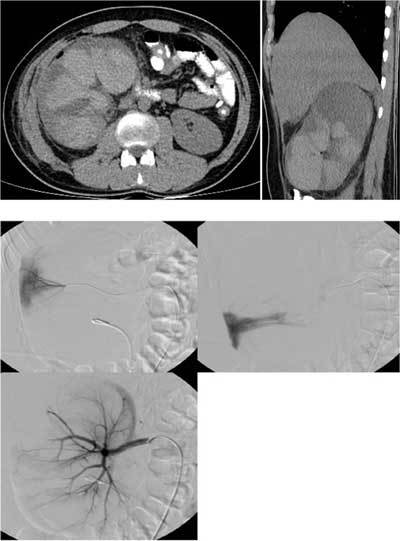


The patient was discharged from the Urology department three days after the endovascular procedure and contacted by phone for follow-up.

## Comment

Percutaneous nephropyelolithotomy (PCNL) is considered the standard procedure for staghorn and large-volume renal calculi, as well as upper tract calculi that cannot be treated with other modalities, difficult lower pole stones, cystine nephrolithiasis, and calculi in anatomically abnormal kidneys. PCNL is typically a very safe and well-tolerated procedure. But it has general and specific sets of complications, as well. Bleeding, pseudoaneurysm, diaphragmatic and associated organ injuries, systemic inflammatory response syndrome/sepsis, renal collecting system injury, renal dysfunction, and death can be counted as the common postoperative complications.

Acute hemorrhage after PCNL due to injury to the great vessels or main kidney vessels is a rare entity. Multiple renal punctures, upper pole renal access, an inexperienced surgeon, a solitary kidney, and staghorn calculus all significantly increase the risk of major bleeding. Postoperative tachycardia, hypotension, abdominal and/or flank pain/tenderness, and gradually decreasing hematocrit and hemoglobin values are the warning symptoms of probable active bleeding. Most vascular injuries occur during initial percutaneous access. Due to the proximity of large vessels and paucity of renal parenchyma to provide tamponade, major bleeding during direct access to renal pelvis is more commonly seen. Although bleeding with initial access and tract dilatation is often venous in nature arising from the percutaneous tract, renal capsule or parenchyma, it can also be arterial as in our case. Mild to moderate bleeding can often be controlled by tamponade with a balloon dilator or Kaye tamponade catheter. Placement of a larger nephrostomy tube, appropriate intravenous hydration, intravenous administration of mannitol, and blood transfusion in necessary situations can also be beneficial. Significant perioperative renal hemorrhage requiring intervention is quite rare and pseudoaneurysm is the most common pathology at angiography. Treatment can be successfully achieved with superselective angioembolization. This specific procedure can also be effectively used for active renal arterial hemorrhages as in our case.

## Competing Interests

The authors declare that they have no competing interests.
